# Landscape of the gut archaeome in association with geography, ethnicity, urbanization, and diet in the Chinese population

**DOI:** 10.1186/s40168-022-01335-7

**Published:** 2022-09-13

**Authors:** Xiaowu Bai, Yang Sun, Yue Li, Maojuan Li, Zhirui Cao, Ziyu Huang, Feng Zhang, Ping Yan, Lan Wang, Juan Luo, Jing Wu, Dejun Fan, Hongxia Chen, Min Zhi, Ping Lan, Zhong Zeng, Xiaojian Wu, Yinglei Miao, Tao Zuo

**Affiliations:** 1 Guangdong Institute of Gastroenterology, The Sixth Affiliated Hospital of Sun Yat-sen University, Sun Yat-sen University, Guangzhou, Guangdong China; 2 Center for Fecal Microbiota Transplantation Research, The Sixth Affiliated Hospital of Sun Yat-sen University, Sun Yat-sen University, Guangzhou, Guangdong China; 3 Department of Colorectal Surgery, The Sixth Affiliated Hospital of Sun Yat-sen University, Sun Yat-sen University, Guangzhou, Guangdong China; 4 Department of Gastroenterology, The First Affiliated Hospital of Kunming Medical University, Kunming, Yunnan China; 5 Yunnan Province Clinical Research Center for Digestive Diseases, Kunming, Yunnan China; 6 Department of Gastrointestinal Endoscopy, The Sixth Affiliated Hospital of Sun Yat-sen University, Sun Yat-sen University, Guangzhou, Guangdong China; 7 Department of Gastroenterology, The Sixth Affiliated Hospital of Sun Yat-sen University, Sun Yat-sen University, Guangzhou, Guangdong China; 8 Department of Organ Transplantation Center, The First Affiliated Hospital of Kunming Medical University, Kunming Medical University, Kunming, Yunnan China

**Keywords:** Archaea, Gut, Geography, Ethnicity, Urbanization, Diet

## Abstract

**Background and aims:**

The human gut is home to a largely underexplored microbiome component, the archaeome. Little is known of the impact of geography, urbanization, ethnicity, and diet on the gut archaeome in association with host health. We aim to delineate the variation of the human gut archaeome in healthy individuals and its association with environmental factors and host homeostasis.

**Methods:**

Using metagenomic sequencing, we characterized the fecal archaeomes of 792 healthy adult subjects from 5 regions in China, spanning 6 ethnicities (Han, Zang, Miao, Bai, Dai, and Hani), consisting of both urban and rural residents for each ethnicity. In addition, we sampled 119 host variables (including lifestyle, diet, and blood parameters) and interrogated the influences of those factors, individually and combined, on gut archaeome variations.

**Results:**

Population geography had the strongest impact on the gut archaeome composition, followed by urbanization, dietary habit, and ethnicity. Overall, the metadata had a cumulative effect size of 11.0% on gut archaeome variation. Urbanization decreased both the α-diversity (intrinsic microbial diversity) and the β-diversity (inter-individual dissimilarities) of the gut archaeome, and the archaea-to-bacteria ratios in feces, whereas rural residents were enriched for *Methanobrevibacter smithii* in feces. Consumption of buttered milk tea (a characteristic diet of the rural Zang population) was associated with increased abundance of *M. smithii*. *M. smithii* was at the central hub of archaeal-bacterial interactions in the gut microecology, where it was positively correlated with the abundances of a multitude of short chain fatty acid (SCFA)-producing bacteria (including *Roseburia faecis*, *Collinsella aerofaciens*, and *Prevotella copri*). Moreover, a decreased abundance of *M. smithii* was associated with increased human blood levels of cholinesterase in the urban population, coinciding with the increasing prevalence of noncommunicable diseases (such as dementia) during urbanization.

**Conclusions:**

Our data highlight marked contributions of environmental and host factors (geography, urbanization, ethnicity, and habitual diets) to gut archaeome variations across healthy individuals, and underscore the impact of urbanization on the gut archaeome in association with host health in modern society.

Video Abstract

**Supplementary Information:**

The online version contains supplementary material available at 10.1186/s40168-022-01335-7.

## Introduction

Archaea, the third domain of life, is increasingly recognized as a pivotal component of the human gut microbiota, co-existing with the commensal bacteria in the gut [1]. Advances in next-generation sequencing have greatly expanded our understanding of the taxonomic and genomic diversity of archaea, though a limited number of archaea have been isolated and cultured [2]. The ecology of archaeome was extensively studied in various environments. However, the archaeome inhabiting the gastrointestinal (GI) tract of humans was substantially under-studied. While methanogenic archaea were revealed to be a prominent part of the human gut archaeome [3], particularly in western populations [1, 4, 5], little is known about the diversity of methanogens and other archaeal lineages, and their associations with human health at a large population level in the Chinese population.

The gut bacterial microbiome (bacteriome) was known to be highly diverse and individual-specific, varying across geography, ethnicity, lifestyle, and diet [6–10]. However, it is unclear about the impact of environmental and host factors, including geography, ethnicity, and diet, on the configurations and variations of the gut archaeome in average, healthy populations. Urbanization is posited to deplete gut bacterial species in humans, linking modern lifestyles to decreased bacterial microbiome diversity and a predisposition to western conditions including obesity and autoimmune diseases, such as inflammatory bowel disease (IBD) [11–13]. However, the impact of environment and urbanization on the human gut archaeome is also unclear. We, therefore, hypothesize that the gut archaeaome in humans is highly heterogeneous across healthy individuals and is substantially influenced by these metadata variables including both environmental and host factors. Beyond that, the interactions between gut archaea and bacteria as well as their functional links to host (patho) physiology remain largely under-explored. Our prior studies of the bacterial, viral and fungal fractions of the gut microbiome in healthy Chinese found substantial trans-kingdom interactions between various microbial species as well as microbial-host health associations [14, 15]. As another integral member of the gut microbiome, archaea are also posited to play crucial roles in the gut microbial ecology underlying human health. Overall, the complex archaeal community represents one of the biggest gaps in our understanding of the human microbiome.

**Fig. 1:**
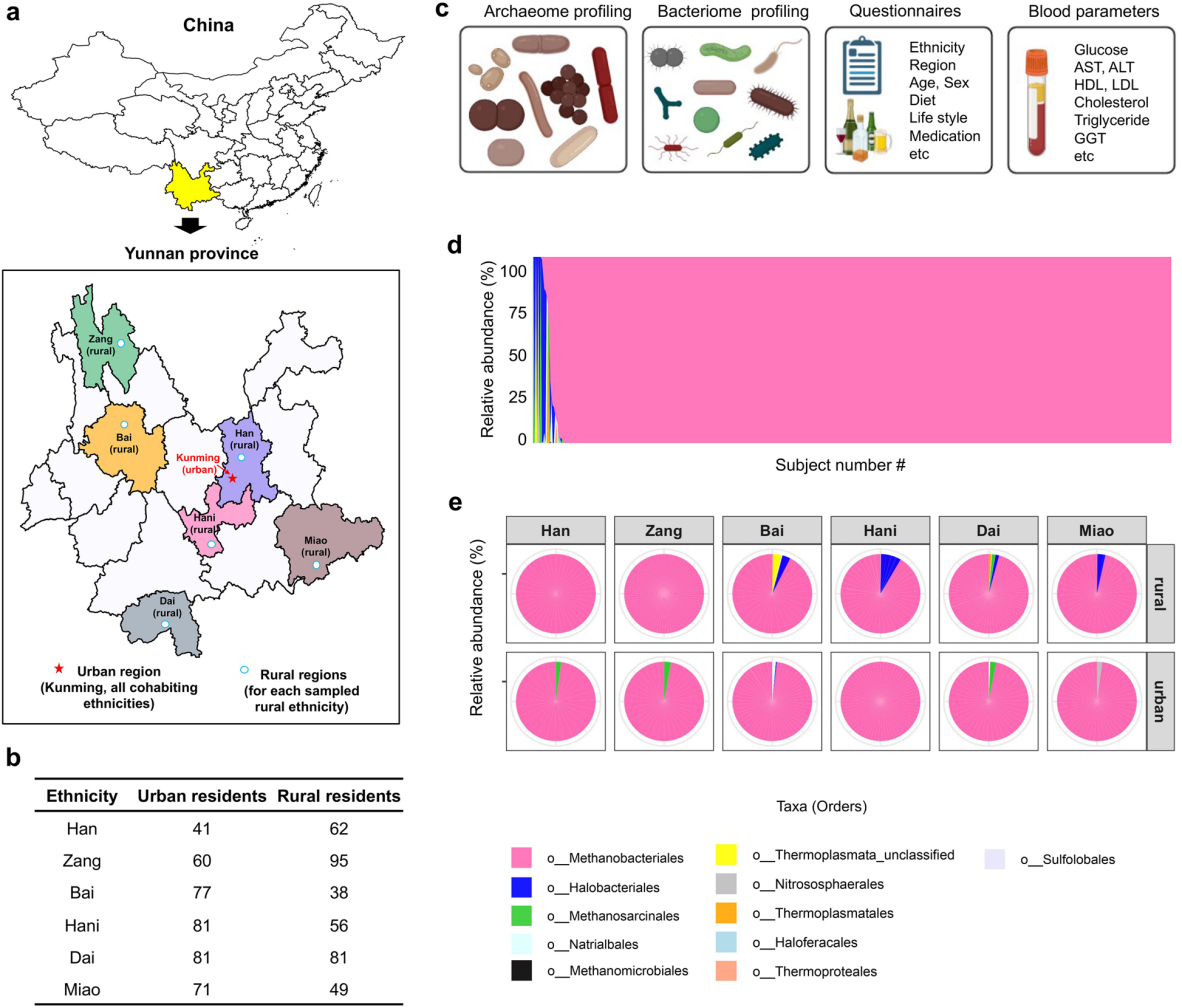
Variations in gut archaeome across healthy individuals. **a** Geographical distribution of the studied Chinese populations. Yunnan Province was the sampling region. Urban individuals were recruited and sampled from the Kunming City (the provincial capital of Yunnan Province), consisting of individuals from 6 ethnicities (Han, Zang, Miao, Bai, Dai, and Hani). Rural populations corresponding to each ethnicity were individually recruited and sampled at each rural region circumferential of Kunming. **b** Number of subjects recruited in this study (*n* = 792). **c** An overview of study design and data collection regime, including archaeome and bacteriome profiling, metadata questionnaire investigation, diet record (food frequency questionnaire) investigation, and blood biochemical measurements. **d** Variations in gut archaeome composition at the order level across all study subjects, plotted according to the ranking abundance of archaeal orders. **e** Order-level gut archaeome compositions plotted according to ethnicity (Han, Zang, Miao, Bai, Dai, and Hani) and residency (rural versus urban)

Herein, we depict gut archaeome variations in association with geography, ethnicity, urbanization, diet, and host blood measurements with functional implications, in hitherto the largest cohort of populations of varied ethnic backgrounds who dwell in regions with different levels of urbanization in China. Fecal samples were collected from a total of 792 healthy Chinese individuals from the most geographically and ethnically diverse province in China, the Yunnan Province (6 ethnicities were enrolled: Han, Zang, Miao, Bai, Dai, and Hani) (median age 36 years, interquartile range [IQR] 22–43 years, Fig. 1a, b, Supplementary Tables 1 and 2). Ultra-deep fecal shotgun metagenomic sequencing (average sequence depth, 55 ± 7 million pair-end reads per sample) was performed on those individuals to simultaneously investigate the archaeome and the bacteriome. Yunnan is a large province in China consisting of both urban and rural residents, where Kunming (the provincial capital of Yunnan) is an urban city with populations from different ethnicities (mostly ethnic Han, the majority ethnicity in China) (Fig. 1A). Each minority ethnic group residing in Kunming originates from disparate rural districts in Yunnan, therefore both the urban residents in Kunming and the corresponding rural-district residents were recruited for each studied ethnicity (Fig. 1a, b). In parallel to fecal sampling, we collected subject metadata including environmental exposures, lifestyle, diet records, host biological traits medications, and blood biochemical measurements (consisting of 119 variables, Fig. 1c, Supplementary Tables 2, 3, and 4). The unique population distribution and comprehensive phenotype data allowed us to dissect the impact of geography, ethnicity, urbanization, diet, and host-related factors on gut archaeome configuration, as well as to generate functional insights from archaea-blood parameter correlations, in a well-defined healthy Chinese population.

## Results

### The landscape and variations of the gut archaeome across healthy Chinese populations

The landscape of the fecal archaeome of profiled Chinese individuals formed a compositional continuum at both the order and species levels (Fig. 1D; Supplementary Figure 1A), suggesting a large variation in the gut archaeome composition across healthy individuals. The fecal archaeome of healthy Chinese individuals was predominated by the order Methanobacteriales (present in 93.9% of subjects with a relative abundance > 99%, Fig. 1D), where *Methanobrevibacter smithii, Methanobrevibacter oralis,* and *Methanosphaera stadtmanae* (all are members of Methanobacteriales) were the most abundant species, prevalent in 70.7%, 43.6%, and 33.1% respectively amongst healthy individuals (Supplementary Figure 1B, C). Population ethnicity and rural versus urban residency both had significant effects on the fecal archaeome configuration (permutational multivariate analysis of variance [PERMANOVA]: both false discovery rate [FDR] adjusted *p* < 0.05; effect size *R*^*2*^ 0.026 and 0.018 respectively for the effects of ethnicity and rural versus urban residency), where the populations displayed ethnicity- and rural/urban residency-dependent variations in Methanobacteriales, Halobacteriales, and Methanosarcinales (Fig. 1E).

Methanomassillicoccales, an order of archaea previously reported to be prevalent in western populations [1, 16], was not present in our cohort (Fig. 1D,E). We then compared the prevalence of Methanomassillicoccales in the Chinese populations versus western populations; we additionally downloaded publicly available fecal metagenomic datasets for 6 external cohorts for comparative archaeome analysis, including 2 Chinese populations from Beijing (*n* = 161) and Guangzhou (*n* = 171), and 4 western populations from HMP (*n* = 239), Irish (*n* = 117), the UK (*n* = 211), and the USA (*n* = 97). Consistently, Methanomassillicoccales was not present in the Chinese populations (including Yunnan, Beijing, and Guangzhou) whereas was present in the interrogated western populations (*one way anova*, all Holm-Bonferroni adjusted *p* < 0.01, Supplementary Figure 1D, E, mean prevalence of Methanomassillicoccales in the Chinese versus western populations: 0% versus 3.9%), despite variations in DNA extraction and metagenomic sequencing protocols between studies. This trans-national archaeome data highlights a large heterogeneity in the gut archeaome configuration across the global populations. Taken together, these data indicate that the human gut archaeome is highly variable across average healthy individuals, and that ethnicity and rural/urban lifestyles contribute largely to the compositional variations in the human gut archaeome.

### Host factors associated with gut archaeome variations

**Fig. 2:**
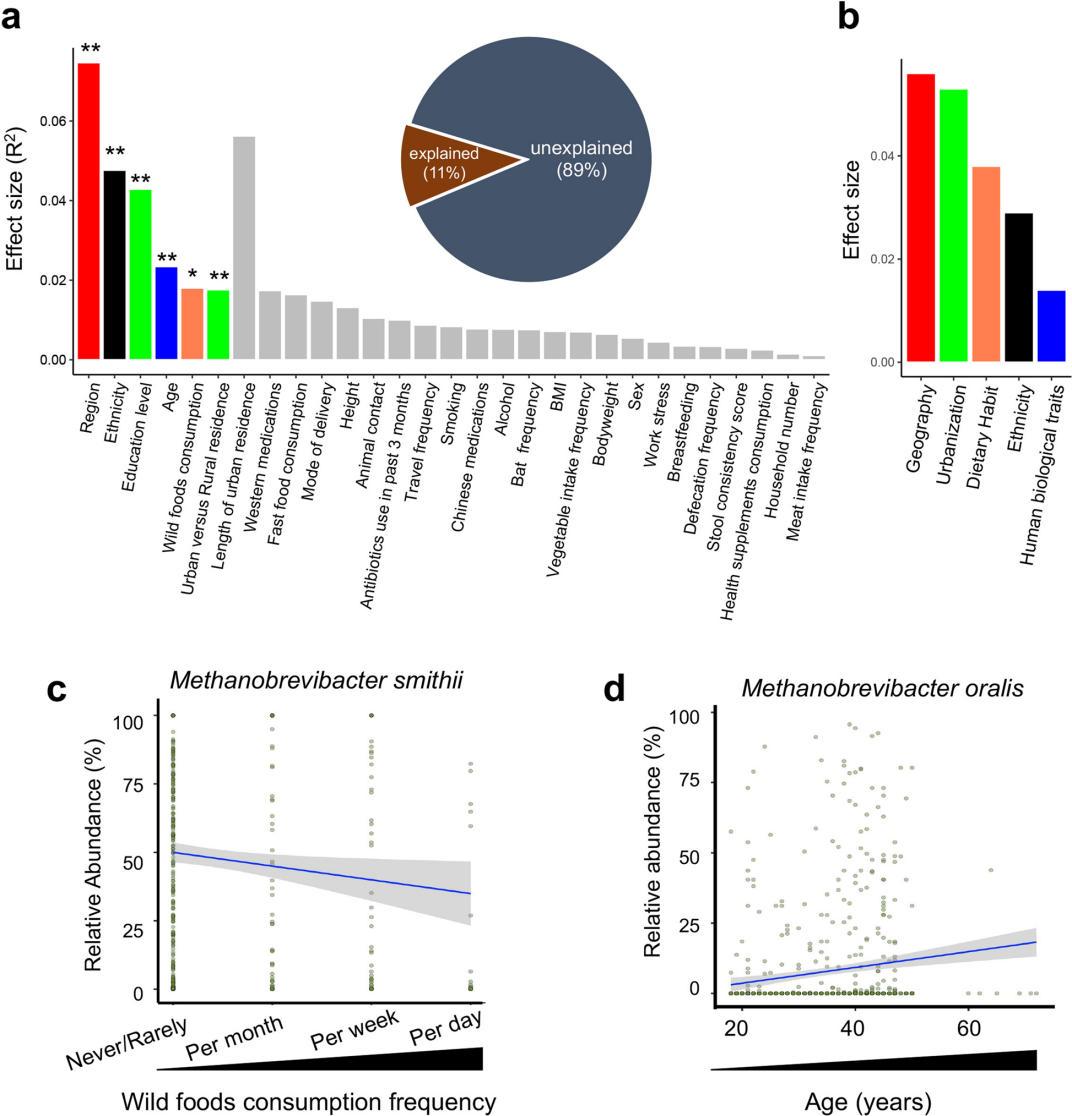
Covariates of the gut archeaome variation and their effect sizes. **a** The effect size of metadata variables in the gut archaeome variation. Archaeome covariates were identified via envfit (vegan) and those with statistical significance (FDR adjusted *p* < 0.05) were colored based on the metadata category shown in **b**. *p* < 0.05*, *p* < 0.01**. Pie chart shows the fraction of archaeome variation explained by all captured metadata variables. **b** Combined effect size of archaeome covariates pooled in predefined categories with covariate distance-based selection. **c** Correlation between relative abundance of *Methanobrevibacter smithii* and frequency of wild foods consumption (graded into 4 continuous levels). **d** Correlation between relative abundance of *Methanobrevibacter oralis* and host age by linear regression

Based on subject phenotyping, we tested 33 metadata variables (Supplementary Table 2) to identify host factors significantly associated with gut archaeome composition. Six factors (geographic region, ethnicity, education level, age, wild foods consumption, and rural/urban residence) were found to have marked effects on the fecal archaeome composition (envfit analysis, all FDR adjusted *p* < 0.05, Fig. 2A). All the interrogated host factors (*n* = 33) combined explained 11.0% of archaeome variations (Fig. 2A), suggesting an additional contribution from unknown factors, stochastic effects, and/or biotic interactions [7, 14]. We then grouped all host factors into 6 predefined categories (geography, urbanization, ethnicity, dietary habit, human biological traits, and medication, see “Methods” section and Supplementary Table 2) and assessed the combined effect size of each category, after removing intrinsic redundant co-linear variables. Overall, geography, urbanization, dietary habit, ethnicity, and human biological traits showed a significant impact on the fecal archaeome composition in a descending order of effect size (bioenv analysis, all FDR adjusted *p* < 0.05, Fig. 2B). Among them, geography had the largest effect size in impacting the fecal archaeome variation, accounting for 5.6% of the archaeome variations (Fig. 2B). Of the geography-related factors (longitude, latitude, and altitude), a western longitude (E) was associated with a decreased relative abundance of *M. smithii* and *M. oralis*, whilst a higher altitude was also associated with a decreased relative abundance of *M. oralis* (via MaAsLin2 analysis, all FDR adjusted *p* < 0.05, Supplementary Fig. 2). Urbanization surrogates (including education level, wild foods consumption, and rural/urban residence) together had an explanatory power of 5.3% on the archaeome composition variations. We next particularly determined the species associated with consumption of wild foods (plants and animals harvested from the wild as opposed to the market or farm) and host age, given their significant impacts on the fecal archaeome composition (Fig. 2A). More frequent wild foods consumption was associated with a decreased relative abundance of *M. smithii* in the fecal archaeome (MaAsLin2 analysis, FDR adjusted *p* < 0.05, Fig. 2C). Increased host age was associated with a higher relative abundance of *M. oralis* in the fecal archaeome (MaAsLin2 analysis, FDR adjusted *p* < 0.05, Fig. 2D). Owning to that a small proportion of the enrolled subjects had a medication history in the preceding 3 months prior to sampling (0.6% had pharmaceutically synthesized drugs and 5.0% had traditional Chinese drugs), no significant effects of medication on fecal archaeome variations were observed (Fig. 2A).

### Urbanization-, geography-, and ethnicity-specific compositions of gut archaeome

**Fig. 3:**
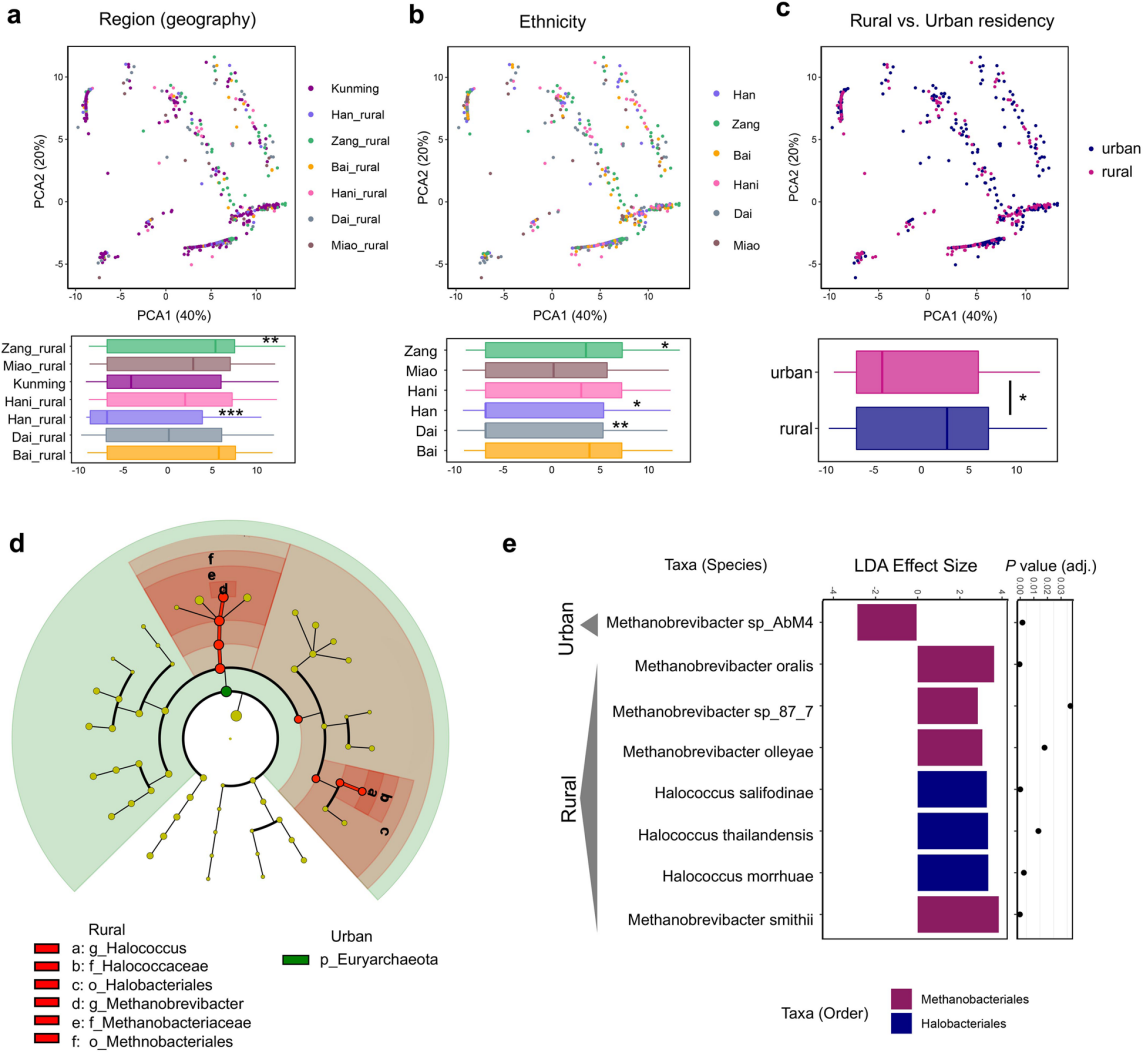
Gut archaeome variations with geography, ethnicity, and rural versus urban residency. The archaeomes were analyzed and plotted via principal component analysis (PCA) based upon the itchison distance between species-level archaeome compositions. **a**, **b** Archaeome variations with geographic region and ethnicity respectively. Comparisons of subject archaeome distribution on PC1 between the base mean of each group of interest (region or ethnicity) and that of all groups. Statistical significance was determined by Wilcoxon rank sum test with Holm-Bonferroni adjustment of *p* values, *p < 0.05, **p < 0.01, ***p < 0.001. **c** Archaeome variations between rural and urban subjects. Comparison of subject archaeome distributions on PC1 between rural and urban residents and statistical significance were determined by *t* test, *p < 0.05. For box plots, the boxes extend from the 1st to 3rd quartile (25th to 75th percentiles), with the median depicted by a horizontal line. **d**, **e** Differentially enriched gut archaeal taxa between rural and urban residents were determined by *LefSE* analysis (only those differential taxa with FDR adjusted *p* values < 0.05 and LDA effect size > 2 are shown). In **d**, taxa color-coated in red denote taxa enriched in rural subjects, while those color-coated in green denote taxa enriched in urban subjects. **e** The discriminatory species between rural and urban archaeomes

To assess the variation of gut archaeome with geography, ethnicity, and urbanization, we performed principal component analysis (PCA) on the archaeal species-level community profiles as a function of region, ethnicity, and rural versus urban residency, respectively (Fig. 3A–C). Individuals from the Zang(rural) and Han(rural) regions showed the most distinct archaeome configurations from other populations residing in the other regions of Yunnan (PERMANOVA, Holm-Bonferroni adjusted *p* < 0.01 and < 0.001 respectively, *R*^2^ 0.17, as reflected on the PC1 axis, Fig. 3A). We subsequently identified region-specific archaeal species by MaAsLin2 (controlling for ethnicity, Supplementary Figure 3). *M. smithii* was specifically enriched in the Zang(rural) group whereas underrepresented in the Han(rural) group (both Holm-Bonferroni adjusted *p* < 0.05, Supplementary Figure 3).

Upon investigating the variation of the gut archaeome composition with population ethnicity, we found that the fecal archaeome of the ethnicities Zang, Han, and Dai differed remarkably from that of other ethnic groups (PERMANOVA, Holm-Bonferroni adjusted *p* < 0.05, < 0.05, and < 0.01 respectively, *R*^2^ 0.20, as reflected on the PC1 axis, Fig. 3B). To determine ethnicity-associated archaeal species, we performed MaAsLin2 analysis on the archaeome composition of the 6 ethnic groups. *M. stadtmanae* was specifically enriched in the ethnic Hani group (Holm-Bonferroni adjusted *p* < 0.05, Supplementary Figure 4A), whilst *M. oralis* was specifically enriched the ethnic Han group (Holm-Bonferroni adjusted *p* < 0.05, Supplementary Figure 4B). *M. smithii* was significantly underrepresented in the ethnic Dai group yet enriched in the ethnic Zang group (Holm-Bonferroni adjusted *p* < 0.05 and < 0.01, respectively, Supplementary Figure 4C), displaying ethnicity-specific gut archaeome signatures.

The fecal archaeome significantly differed between rural and urban residents (all ethnicities combined; *t* test on the dispersion of rural versus urban archaeomes along the PC1 axis, *p* < 0.05, Fig. 3C), and rural/urban residency contributed 1.76% to the overall fecal archaeome variations (envfit analysis, *p* < 0.01, Fig. 2A). To identify fecal archaeal taxa associated with rural or urban residence, we performed LefSE (Linear discriminant analysis Effect Size) analysis on the fecal archaeomes between the rural and urban residents. While *Methanobrevibacter sp_AbM4* was enriched in urban residents, 7 other species (*M. oralis*, *Methanobrevibacter sp_87_7*, *Methanobrevibacter olleyae*, *Halococcus salifodinae*, *Halococcus thailandensis*, *Halococcus morrhuae*, *M. smithii*) were enriched in rural residents (from the orders Methanobacteriales and Halobacteriales, all FDR adjusted *p* < 0.05, Fig. 3E). Overall, more archaeal taxa were enriched in rural residents compared to urban residents (Fig. 3D, E), suggesting that depletion of archaeal taxa in the human gut may be a characteristic of urban living.

### Diversity of gut archaeome across populations

**Fig. 4:**
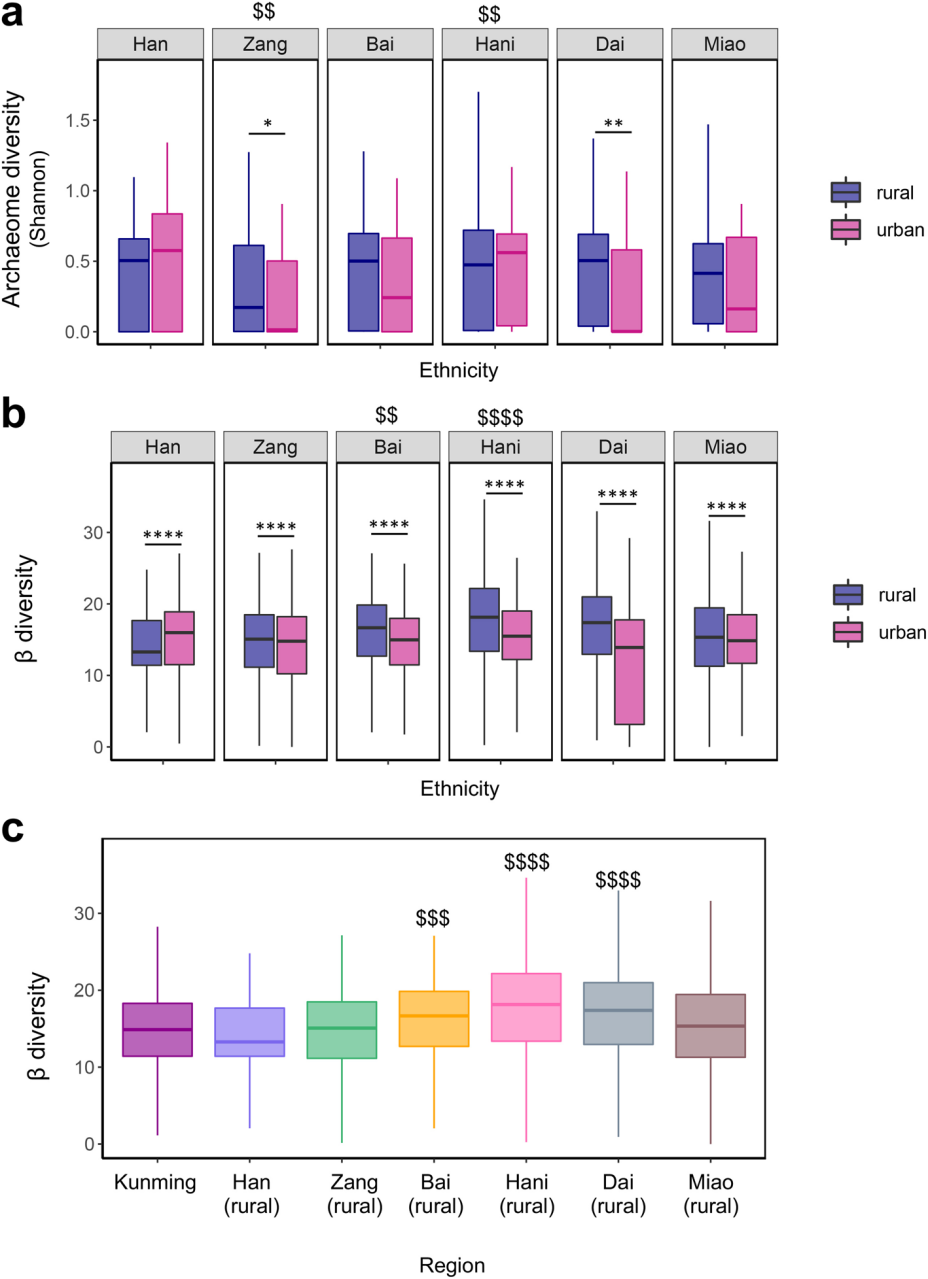
Variations in the α (intra-individual) and β (inter-individual) diversity of the gut archaeome with geography, ethnicity, and rural versus urban residency. **a** The fecal archaeome α diversity, as measured in Shannon diversity index, was plotted and compared across all ethnicities, and between rural and urban subjects with respect to each ethnic group. Across-population comparisons were conducted between the base mean of group of interest and that of all groups, statistical significance was determined by Wilcoxon rank sum test with Holm-Bonferroni adjustment of *p* values, ^$$^*p* < 0.01. Statistical significance between rural and urban populations for each individual ethnic group was determined by Mann-Whitney test, *p < 0.05, **p < 0.01, ***p < 0.001, ****p < 0.0001. **b**, **c** The β diversity of fecal archaeomes with ethnicity and rural versus urban residency (**b**), and geographic region (**c**). The β diversity of archaeomes was calculated as the Bray-Curtis dissimilarities between individual archaeomes. Across-population comparisons were conducted between the base mean of group of interest and that of all groups, statistical significance was determined by Wilcoxon rank sum test with Holm-Bonferroni adjustment of *p* values, ^$$^*p* < 0.01, ^$$$^*p* < 0.001, ^$$$$^*p* < 0.0001. Statistical significance between rural and urban populations for each individual ethnic group was determined by Mann-Whitney test, ****p < 0.0001. For box plots, the boxes extend from the 1st to 3rd quartile (25th to 75th percentile), with the median depicted by a horizontal line

Urbanization has been associated with a decrease in bacterial microbiome diversity, a characteristic of the increase of chronic diseases worldwide [11, 12, 17]. We hence assessed the α diversity (Shannon diversity) of the gut archaeome in association with rural/urban residency and ethnicity. The archaeome species diversity (α diversity) was significantly decreased in urban residents of the ethnicities Zang and Dai, compared to their rural counterparts (*Mann-Whitney* test, *p* < 0.05 and < 0.01 respectively, Fig. 4A). These data ubstantiated the finding that multiple taxa were decreased in urban versus rural residents (Fig. 3D, E), together suggesting that urbanization may ecologically decrease the archeaome richness and diversity in human gut, which may be partly ascribed to higher standards of hygiene and sanitation measures adopted in urban societies.

With regard to ethnicity, the ethnicity Zang exhibited the lowest archaeome diversity across all ethnicities (*one-way anova*, Holm-Bonferroni adjusted *p* < 0.01, Fig. 4A). However, the ethnicity Hani exhibited the highest archaeome diversity amongst all ethnicities (*one-way anova*, Holm-Bonferroni adjusted *p* < 0.01, Fig. 4A). These data imply that ethnicity-specific lifestyles may shape the α diversity of the gut archaeome, analogous to our prior findings reported for the gut bacterial microbiome and virome [14, 15].

We then investigated the β-diversity of the gut archaeome (inter-individual archaeome dissimilarity) in association with rural/urban residency and ethnicity. In contrast to the majority ethnicity Han, the urban residents of which displayed increased β-diversity than its rural counterparts, the urban residents of all other minority ethnicities (Zang, Bai, Hani, Dai, and Miao) showed decreased β-diversity compared to their rural counterparts (*Mann-Whitney* test, all *p* < 0.0001, Fig. 4B). This data indicates that urbanization may homogenize the gut archaeome composition between minority ethnic populations, leading to decreased inter-individual archaeome dissimilarity among them, whereas the multi-ethnicity cohabitation practice in the urban region (Kunming) may increase the inter-individual archaeome dissimilarity among the Chinese Han population. Overall, the populations of Bai(rural), Hani(rural) and Dai(rural) had a significantly higher β-diversity of the gut archaeome than populations residing in other Yunnan regions (*one way anova*, Holm-Bonferroni adjusted *p* < 0.001, < 0.0001, and < 0.0001 respectively, Fig. 4C), suggesting that these populations may have limited archaeal species transmission or exchange between individuals.

### The impact of diet on fecal archaeome composition

**Fig. 5:**
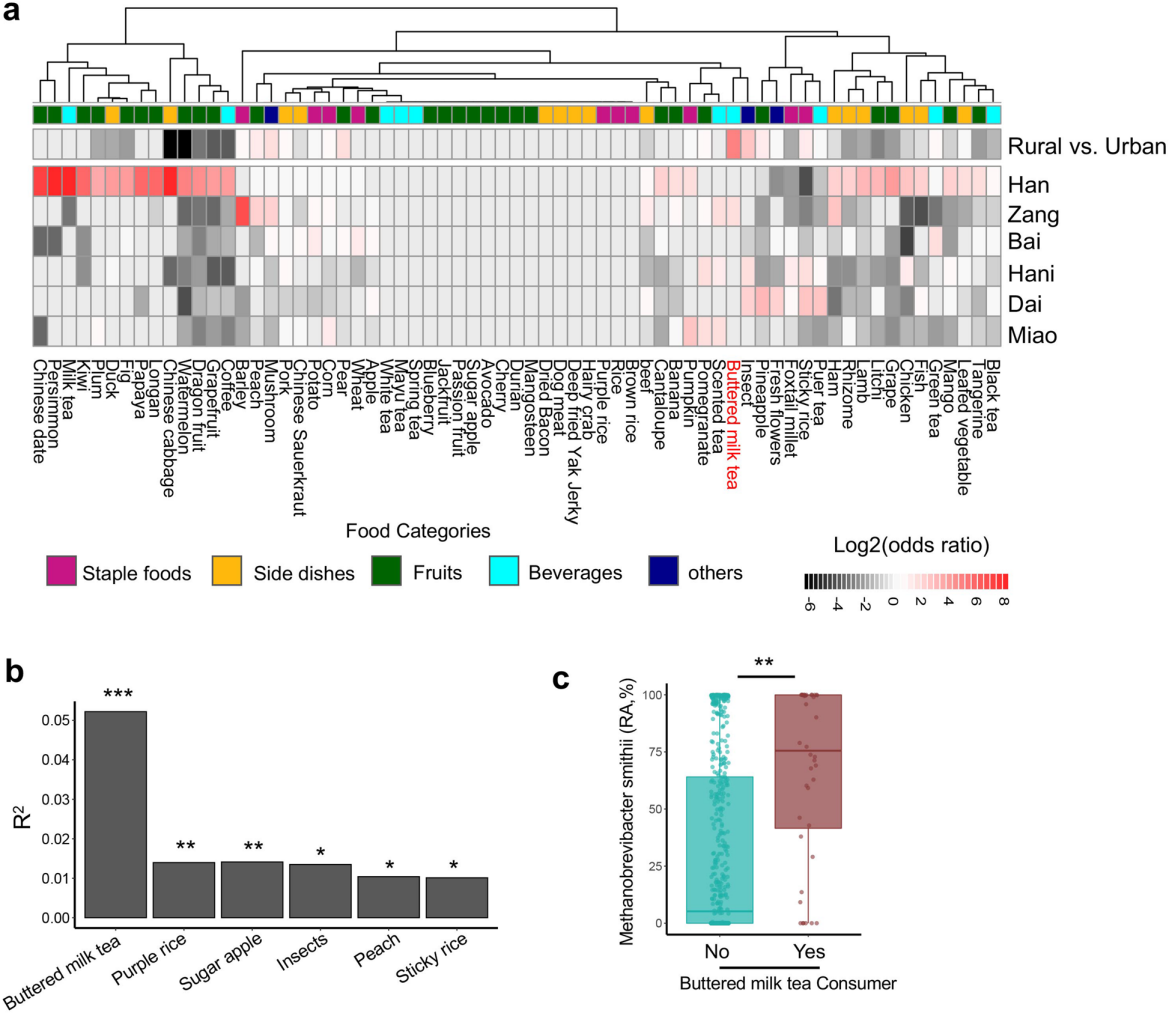
Dietary differences across populations and their effects on gut archaeome variations. **a** Differential consumption patterns of various food items between Chinese populations (rural versus urban populations and different ethnic populations). Odds ratio of likelihood of dietary component within factors of interest was calculated by unconditional maximum likelihood estimation (Wald). Only significant associations (*p* < 0.05) were plotted and colored according to log2 transformed odds ratio. **b** The effect size of food items on variations of the archaeome composition across Chinese populations. Food items were sorted according to their effect sizes. Only those with statistical significance (FDR adjusted *p* < 0.05) were plotted, ***p < 0.001, **p < 0.01, *p < 0.05. **c** The relationship between fecal abundance of *M. smithii* and food consumption of buttered milk tea. Statistical significance between consumers and non-consumers of buttered milk tea was determined by Mann-Whitney test, **p < 0.01. For box plots, the boxes extend from the 1st to 3rd quartile (25th to 75th percentile), with the median depicted by a horizontal line

Following the observation that dietary habits contributed significantly to the gut archaeome variations, explained 3.8% of the overall variations (bioenv analysis, FDR adjusted *p* < 5%, Fig. 2B), we conducted detailed diet recording and interrogated the effect of each food item on archaeome variations. A total of 68 food items (dietary components), characteristic of atypical Chinese foods comprising staple foods, side dishes (mostly cooked meats and vegetables), fruits, and beverages, were included (Supplementary Table 3). Collectively, the dietary structure exhibited marked ethnicity-specific food intake predilections and discriminatory dietary features between rural and urban residents, differing primarily in consumption of fruits, side dishes, and minority ethnicity-specific foods (such as buttered milk tea, insects, barley, pu’er tea, sticky rice) (Fig. 5A).

Among those food items, buttered milk tea, purple rice, sugar apple, insects, peach, and sticky rice showed the most significant effects on the fecal archaeome composition (bioenv analysis, all FDR adjusted *p* < 0.05, Fig. 5B). By MaAsLin2 analysis, a total of 16 associations between archaeal species and specific food item consumptions were identified (FDR adjusted *p* < 0.05, Supplementary Table 5). Among the interrogated food items, consumption of buttered milk tea, which was uniquely consumed by the ethnic Zang (especially rural Zang) population as compared to other Chinese populations (Fig. 5A), showed a significant positive correlation with the relative abundance of *M. smithii* (*Mann-Whitney* test between consumers and non-consumers of buttered milk tea, *p* < 0.01, Fig. 5C). The ethnic Zang-specific diet buttered milk tea contains complex dietary fibers, which are a class of nutrients metabolically preferred and fermented by *M. smithii* in synergy with other co-inhabiting bacteria in the gut [18–20]. These data partly explained the observations that *M. smithii* was the most significantly enriched in the Zang(rural) population compared to other Chinese populations (Supplementary Figure 3), highlighting a link between dietary component and the gut archaea. In addition, the consumptions of insects and sticky rice were both characteristics of ethnic Dai’s diet (Fig. 5A), which also showed a significant impact on the fecal archaeome composition (Fig. 5B), suggesting their contribution to the prominent difference between the archaeome community structure of the ethnicity Dai and other ethnicities (Fig. 3B). These data together indicate that food options and dietary habits (especially those preferred by minority ethnicities and rural residents) may crucially shape the gut archaeal membership in humans.

### Correlations between archaeome and host blood parameters

**Fig. 6:**
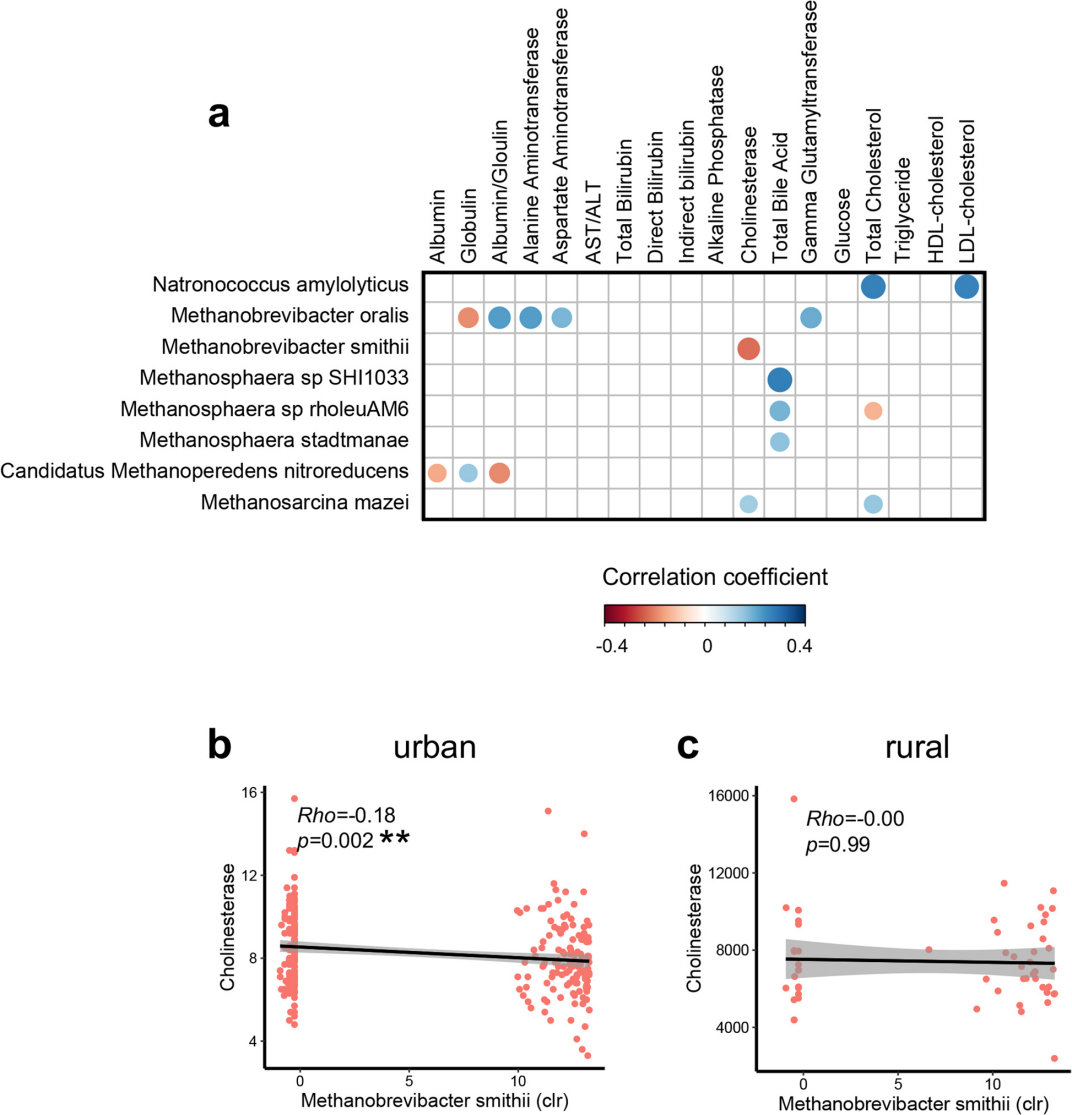
Correlation between gut archaeal species and host blood parameters. **a** Correlations between archaeal species abundance and blood parameters. Correlations between species abundance and blood biochemical measurements were calculated through spearman correlation test with FDR correction. Correlation coefficient was calculated, whilst statistical significance was determined for all pairwise comparisons. Only statistically significant correlations were shown where the correlation dot was color-intensified according to correlation direction (positive or negative) and coefficient size. **b**, **c** Correlations between the fecal abundance of *M. smithii* and the blood level of cholinesterase, viewed via regression plot, in urban (**b**) and rural (**c**) residents respectively. Correlation coefficient *Rho* and statistical significance were calculated by spearman correlation analysis, **p < 0.01

To gain insight into the potential functional link between the gut archaeome and human health, we probed a panel (*n* = 18) of blood parameters in a subset of enrolled subjects who consented to clinical blood measurements (*n* = 561) (Supplementary Table 4). We then investigated the relationship between fecal archaea and host blood parameters (by spearman correlation with FDR correction), which yielded a number (*n* = 17) of significant correlations between fecal archaeal species and blood parameters (shown in Fig. 6A). Of the significant associations, the rural-residency associated species, *M. smithii*, was inversely associated with blood concentrations of cholinesterase (a parameter associated with dementia and cognitive disorders) in urban residents (Fig. 6A, B), though no association was observed for rural residents (Fig. 6C). These data indicate that urbanized lifestyles may calibrate the functional effects of archaea on host health and *M. smithii* may play a beneficial role in counteracting cognitive disorders in urbanized societies.

Interestingly, *M. oralis* showed significant positive correlations with multiple liver pathology-associated blood parameters (Fig. 6A), including aspartate aminotransferase (AST), alanine aminotransferase (ALT) (both parameters known to increase in liver damage [21]), and GGT (a parameter known to increase in metabolic syndrome-associated liver pathologies [21–23]). These data imply that gut *M. oralis* may play a detrimental role in the hepatopathology-related diseases. *M. oralis* is an archaeal species associated with periodontitis [24–26]. In favor of our hypothesis, increasing evidence has indicated that periodontitis participates in the progression of liver diseases [27–29]. These data together underscore the potentially crucial role of gut archaea in human physiology and health, which may extend beyond the gut.

### Trans-domain interactions between gut archaea and bacteria

**Fig. 7:**
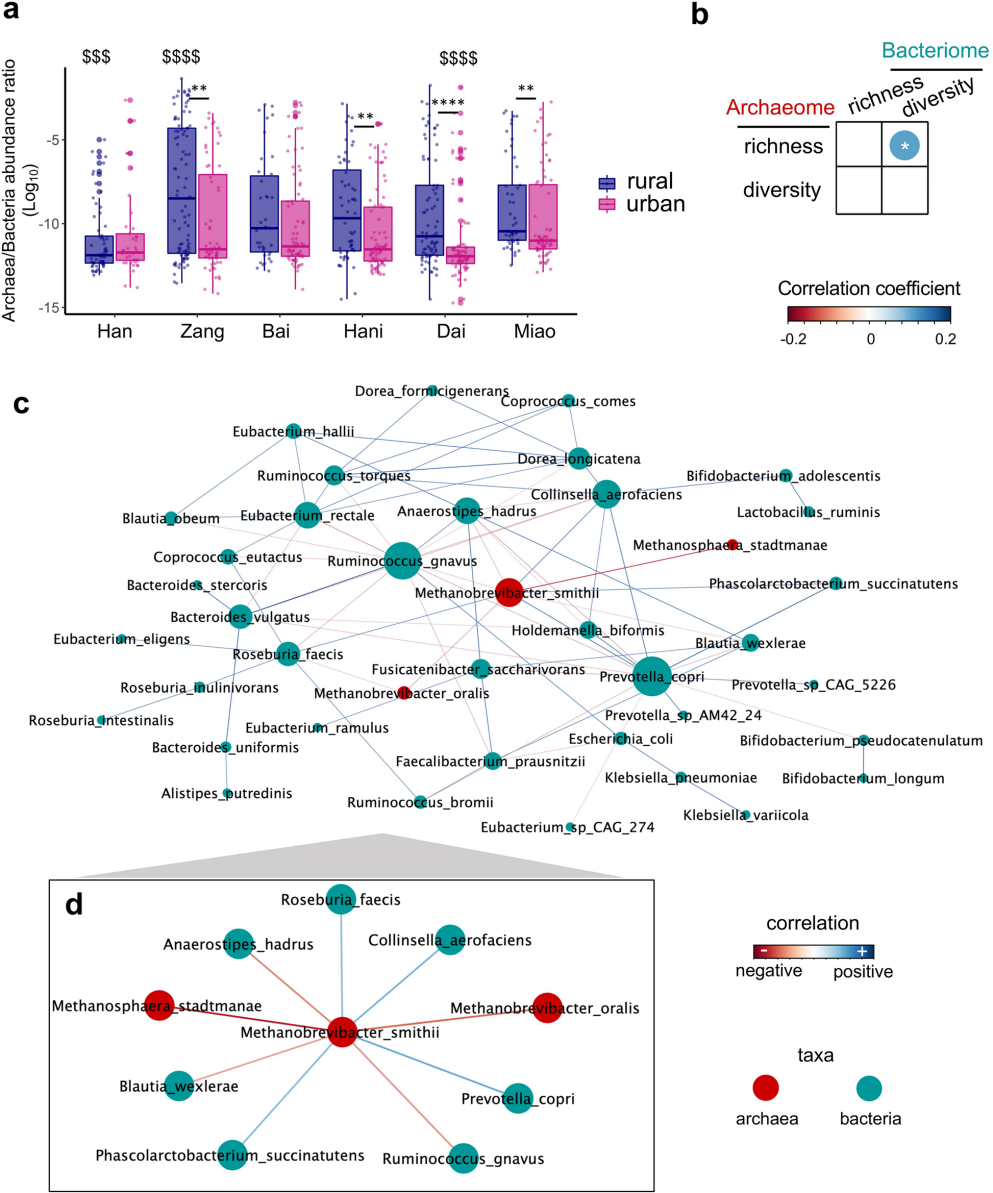
Trans-domain relationships between archaea and bacteria in the gut. **a** Fecal archaea-to-bacteria abundance ratio across populations in association with ethnicity and rural versus urban residency. Across-population comparisons were conducted between the base mean of group of interest and that of all groups, statistical significance was determined by Wilcoxon rank sum test with Holm-Bonferroni adjustment of *p* values, ^$$$^*p* < 0.001, ^$$$$^*p* < 0.0001. Statistical significance between rural and urban populations for each individual ethnicity was determined by Mann-Whitney test, **p < 0.01, ****p < 0.0001. **b** Correlations between the species diversity and richness of the gut archaeome and bacteriome. Pearson correlation tests were performed for coefficient estimation and statistical significance determination, *p < 0.05. **c**, **d** Between-microbial species correlations were calculated via *SparCC*. Only those correlations with |correlation coefficient| > 0.1 and statistical significance (FDR adjusted *p* < 0.05) were plotted and visualized via *Cytoscape*. Each node denotes a microbial species and color-coated according to its taxonomic domain of archaea (darkred) or bacteria (darkgreen). The node size is proportional to the degree of its connectivity to other nodes. Specific microbial species associated with *M. smithii* were plotted in **d** for easier visualization. The connection lines between nodes were color-coated according to correlation direction (positive or negative) and were color-intensified according to coefficient size

The relative abundance of archaea is substantially lower than that of bacteria in feces, with a median archaea-to-bacteria ratio of 0.13% (IQR 0.06–4.31%, Fig. 7A). The fecal archaea-to-bacteria ratios of urban residents of the ethnicities Zang, Hani, Dai, and Miao were significantly lower than that of their rural counterparts (*Mann-Whitney* test, *p* < 0.01, 0.01, 0.0001, and < 0.01 respectively, Fig. 7A). Overall, the Han(rural) and Dai(urban) populations exhibited the lowest archaea-to-bacteria ratios, whereas the Zang(rural) populations had the highest archaea-to-bacteria ratios (*one-way anova*, Holm-Bonferroni adjusted *p* < 0.001, < 0.0001, and < 0.0001, respectively, Fig. 7A). These data suggest that urbanization may have a robust effect on decreasing the relative abundance of archaea in the gut microbiome of minority ethnic populations, while the majority ethnic population (Chinese Han) remains not significantly affected, which may relate to the rural/urban- and ethnicity-specific lifestyles and environments.

We next explored the trans-domain associations between the fecal archaeome and bacteriome. A significant positive correlation was identified between archaeome richness and bacteriome diversity (Spearman correlation *Rho* 0.1, *p* < 0.05, Fig. 7B), suggesting an overall mutualistic relationship between the archaeal and bacterial species in the gut ecosystem. We then analyzed the trans-domain interactions across archaeal and bacterial species based on their abundance profile in feces. Through *SparCC* correlation analysis, we observed both strong trans-domain and intra-domain correlations, which were predominated by the archaeal species *M. smithii* and the bacterial species *Prevotella copri* and *Ruminococcus gnavus* in the ecological network (Fig. 7C and Supplementary Table 6). Among the archaeal species, *M. smithii* had the most abundant associations with other microbial species (Fig. 7C, D). Interestingly, *M. smithii* correlated positively with multiple bacterial species producing short chain fatty acids (SCFAs), including *Roseburia faecis*, *Collinsella aerofaciens*, and *P. copri* (Fig. 7D and Supplementary Table 6). While *M. smithii* was a rural residence associated archaeal species, it showed positive correlations with those SCFAs-producing bacteria (known to counteract metabolic syndromes and IBD, all urbanization-associated diseases) and an inverse association with *R. gnavus* (a bacterial species known to involve in the pathogenesis of IBD) [11, 30, 31] (Fig. 7D). Altogether, these data underpin intricate trans-domain interactions between archaea and bacteria in the gut, which may have joint functions in human health and disease.

### Functionality variations of the gut archaeome across healthy Chinese individuals

We next explored the functional diversity of the gut archaeome across heathy Chinese, by assessing the genetic abundance of the clusters of orthologous protein groups (COG; via EGGNOG database) and of the metabolic pathways (via MetaCyc database). An array of EGGNOG function modules was specificly enriched in the fecal archaeomes of the ethnic Zang population (via MaAsLin2 analysis, Supplementary Table 7a). Most of the enriched EGGNOG modules were proteins involved in lipid transport and metabolism, amino acid transport and metabolism, nucleotide transport and metabolism, translation, ribosomal structure and biogenesis, GTP cyclohydrolase activity, and coenzyme transport and metabolism (Supplementary Table 7a), indicating an enhanced functionality of nutrients biosynthesis and metabolism in the gut archaeome of the ethnic Zang Chinese. A majority of the ethnic Zang-enriched EGGNOG functions were ascribed to the archaeal species *M. smithii*, which was in line with the increased relative abundance of *M. smithii* in the fecal archaeome of the Zang ethnicity versus other Chinese ethnicities (Supplementary Figure 4C). Concordantly, MetaCyc pathway abundance analysis also revealed that multiple biosynthesis pathways were increased in the gut archaeome of the ethnic Zang Chinese compared to other Chinese ethnic groups, including pathways for l-arginine, l-lysine, archaetidylinositol, chorismite, and CDP-archaeol (via MaAsLin2 analysis, Supplementary Table 7b). These data implicate that ethnicity may influence the functionality of the archaeome beyond its impact on the taxonomic composition of the gut archaeome.

We subsequently compared the gut archaeome functionality between rural and urban Chinese. A large panel of EGGNOG modules and MetaCyc pathways were significantly present in higher relative abundance in rural residents than urban residents (via LefSE analysis, Supplementary Table 8). The metabolic pathways enriched in rural residents include biosynthesis pathways for amino acids (particularly essential amino acids: lysine, valine, isoleucine) and nucleotides, pyruvate (one member of SCFAs) fermentation pathways, and CDP-archaeol biosynthesis (Supplementary Table 8b). These data highlight that rural lifestyle may enrich a more diverse gut archaeome functionality than urban lifestyle, akin to the observed higher taxonomic diversity in the gut archaeome of rural residents than urban residents.

## Discussion

The human gut bacterial microbiome is unique to each individual and its composition is primarily influenced by factors such as geography, lifestyle, diet, medication, environment, and genetics [6–10]. However, the gut archaeome is a critical component of the human gut microbiome, its compositional variations at the healthy population level, associations with host factors, and their functional implications are largely unclear. In the present study, we showed the substantial impact of geography, ethnicity and urbanization on human gut archaeome composition using population-based ultra-deep shotgun metagenomics sequencing. Factors related to geography showed the strongest effect, followed by urbanization, dietary habit and ethnicity. Akin to other metadata-bacteriome association studies [7, 8], the profiled host metadata variables together had a cumulative, non-redundant effect size of 11.0% on the gut archaeome variations across healthy individuals. Similarly, all those metadata variables combined explained 15.6% of the gut bacteriome variations, as per our prior report [14]. These data suggest the influence of additional, currently unknown covariates as well as intrinsic microbial ecological factors on gut archaeome variations has yet to be further unveiled. Only a small proportion of the population had medication exposures in the preceding 3 months before sampling, which may result in insignificant effects observed for medicinal variables on the archaeome composition.

Urbanization is one of the major demographic characteristics of the twenty-first century [11, 32]. It is a process indicating transitions from a rural to an urban way of life in modern societies. The rapid urbanization experienced in the developing world has been associated with an increasing incidence of a number of western diseases [11–13]. Urbanization is hypothesized to deplete the gut bacterial species in humans, partly accounting for the uprising non-communicable western diseases [12]. However, it is unknown how urbanization affects the gut archaeome. Our study revealed that urbanization decreased the gut archaeome richness and/or diversity and the archaea-to-bacteria ratios in the gut, potentially another contributor to the increasing prevalence of western diseases during urbanization. Meanwhile, both consumption of buttered milk tea (the minority ethnic Zang-specific diet) and rural residence were associated with increased abundance of *M. smithii* in human gut, indicating that ethnicity-specific and rural lifestyles may shape the gut archaeome configuration. *M. smithii* was reported to be underrepresented in the feces of patients with IBD [31, 33], the incidence of which was increased with urbanization [11]. Consistently, the specific enrichment of *M. smithii* in the rural Zang population is in agreement with our prior epidemiological observation that the incidence of IBD in rural Zang Chinese was markedly lower than that in urban Han Chinese [34]. These data indirectly suggest that *M. smithii* may play a beneficial role in combating IBD.

*M. smithii* plays an important role in the efficient digestion of polysaccharides with complex chemical structures (highly enriched in the buttered milk tea due to the addition of roasted barley), by both optimizing hydrogen levels for bacterial polysaccharide digestion and consuming the end products of bacterial fermentation [18–20]. Interestingly, upon bacteria-archaea correlation analysis, a number of SCFAs-producing bacteria (including *R. faecis*, *C. aerofaciens*, and *P. copri*) which carry out anti-inflammatory functions were found to have positive associations with *M. smithii*, hinting at mutualistic/syntrophic versus antagonistic relationships between gut bacteria and archaea. A prominent example of bacterial-archaeal syntrophism was previously established for *Ruminococcus* and methanogens, where the methanogens consume H_2_, allowing *Ruminococcus* to produce twice as many ATP molecules from the same amount of substrate [35]. From our data, *Prevotella*, *Ruminococcus, Collinsella,* and *Methanobrevibacter* may form a syntrophic guild specified for the degradation of starches and complex polysaccharides contained in rural and ethnic diets, as well as for SCFA generation and depletion of gut H_2_. This is also reflected in our observation that rural residents had an increased abundance of SCFA (pyruvate) fermentation module in the gut archaeome, compared to urban residents. *Prevotella* assists in breaking down carbohydrate/fiber-rich foods into simpler sugars (poly- and monosaccharides) and other products (such as succinate) to be fermented by bacteria such as *Ruminococcus* and *Collinsella* [5, 36, 37]. Fermentation byproducts (including H_2_ and acetate−/H+) produced would then be consumed by *Methanobrevibacter* with the subsequent production of CO_2_ and/or CH_4_ [35, 38, 39]. Altogether, our results highlight a salient 4-unit interplay axis of diet/lifestyle-archaea-bacteria-host, while the causal links and functional implications for human health and disease remain to be established.

There are several shortcomings of our study. First, due to the largely under-discovered and uncultivated nature of human colonized archaea [16, 40, 41], it leads to an underrepresentation of archaeal taxa in the reference archaea database and consequently incomplete profiling of the diversity of fecal archaeal species across populations in the present study. Second, the diet survey was a food questionnaire investigation of past 1-month food items intake, as opposed to a 24-hour recall food frequency questionnaire survey, which makes the dietary record less reflective of recent food consumption and less quantitative in terms of determining nutrients. Third, one crucial biological function of a multitude of methanogenic archaeal species is to produce volatile methane. We were however not able to measure the methane emissions from human breath or feces in real-time when sampling, considering the poor sampling and testing conditions in distant rural regions. It precludes us from methanogenesis analysis on the gut archaeome. Fourth, our results were generated from stool specimens, the presence of archaea in a stool sample does not necessarily indicate that they are colonized in the gut. In order for any archaeon to impact on human health/disease, one would expect a persistent colonization in a specific gut segment. Future studies are encouraged to explore the active presence and functions of archaea in the gut. We acknowledge that some findings of our study are speculative and correlative, future in-depth studies are warranted to unravel the cause versus consequence relationship between gut archaeome and host factors, as well as the downstream health effects and mechanistic insights.

## Conclusions

In summary, our study unveiled the prominent effect of population geography and ethnicity on human gut archaeome compositions. Future research exploring clinical significance and applications of the gut archaeome should be conducted in other populations for precision medicine purposes. Consistent with the prevailing hypothesis that urbanization is associated with depletion of gut bacteria [11, 12], we found that individuals living in urbanized regions had a decreased richness of the gut archaeome. Those archaeal species enriched in rural- and minority ethnicity-populations with specific lifestyles and habitual diets might be exploited for functional studies combating prevalent western diseases. Changes in environment and residency region are inevitable with increasing urbanization and population immigration, which are often associated with risks for certain diseases, such as obesity, childhood allergies, diabetes mellitus, and IBD [42–44]. Given the pathogenesis of such diseases are related to alterations in the gut microbiome [45–48], further studies regarding the functional consequences of disparate archaeome configurations and taxa merit in-depth investigation.

## Methods

### Cohort description and study subjects

A total of 792 healthy Chinese from the Yunnan Province, China, were enrolled from the ethnicities Han, Zang, Miao, Bai, Dai, and Hani (rural and urban residents were included for each ethnic group) (Fig. 1A–C, Supplementary Table 1). The study was approved by the Institutional Review Board (IRB) and the Research Ethics Committee of the First Affiliated Hospital of Kunming Medical School, China (Ref. No.: 2017.L.14). All subjects consented to provide fecal samples, and completed environmental and dietary questionnaires. Written informed consents were obtained from all subjects. Fecal samples from the study subjects were stored at -80°C for metagenomics sequencing followed by archaeome and bacterial microbiome (bacteriome) analyses. Clinical data were obtained by medical practitioners and shown in Supplementary Table 2.

### Dietary record investigation

Dietary questionnaire investigation was conducted by a dedicated dietitian, the result of which was shown in Supplementary Table 3. Dietary questionnaire was designed for Chinese populations, consisting of conventional Chinese foods, ranging from staple foods, side dishes (various types of cooked meats and vegetables), fruits, beverages (Chinese/herbal tea, coffee), and ethnic minority foods in Yunnan (insects, flowers and various types of mushrooms). Intake of these food categories in the recent 1 month was documented as binary. The dietary questionnaire employed in this study was designed based on the 2016 Chinese Dietary Guidelines [49]. Given the large diversity and variety of Yunnan ethnic diets (Dai, Hani, Zang, Bai, Miao, and Han) which is highly different from that consumed by other Chinese populations, we customized our questionnaire, made additional adjustment and added other Yunnan-characteristic food items (including insects, flowers, various types of mushrooms, Deep fried Yak Jerky, Barley, etc.), after consulting with local dietitians. Before the formal employment of this modified questionnaire in this study, the questionnaire went through two rounds of pilot test and validation, where if there is confusion about any items and if respondents have suggestions for possible improvements of the items, the questionnaire was further improved to ensure clarity, informativity, and variation of answers.

### Fasting blood parameter measurements

A majority of the studied Yunnan subjects (*n* = 561) also consented to blood sampling and tests for fasting blood glucose, cholesterol, and other blood parameter measurements (as shown in Supplementary Table 4). Blood glucose as well as other biochemical parameters were fasting blood measurements given that these parameters vary substantially throughout the day. In order to stringently control for the variations, blood measurements were conducted in the morning (6–10 a.m.) after overnight fasting (starting from 8 p.m. the day prior to blood sampling). Both feces and blood samples were collected in the following morning on the same day.

### Stool specimen collection and DNA extraction

Stool sample collection followed a standardized operation procedure (SOP) for all sites. Samples from urban areas were stored within 1 h of collection at – 80 °C freezer and those collected from rural areas were immediately stored in dry ice and transported to the laboratory within 8 h in one batch, followed by storage in – 80 °C freezer. Stool DNA was extracted with modifications to the protocols to increase the yield of archaeal DNA. Approximately 100 mg from each stool sample was prewashed with 1 ml ddH_2_O and pelleted by centrifugation at 13,000×*g* for 1 min. DNA was subsequently extracted from the pellet following efficient lysis by bead beating. The extracted fecal DNA was quantified and quality-controlled by Nanodrop and Agilent 2100 Bioanalyzer, after which DNA was used for ultra-deep metagenomics sequencing via Illumina Novoseq 6000 (Novogen, Beijing, China).

### Profiling of fecal archaeal and bacterial microbiome

Raw sequence reads were filtered and quality-trimmed using *Trimmomatic v0.36* [50] as follows: (1) trimming low quality base (quality score < 20); (2) removing reads shorter than 50 bp; (3) removing sequences less than 50 bp long; (3) tracing and cutting off sequencing adapters. Contaminating human reads were filtered using *Kneaddata* (Reference database: *GRCh38 p12*) with default parameters. Profiling of archaeal and bacterial microbiome was performed via the recently updated pipeline *MetaPhlAn3* by mapping reads to clade-specific markers within its intrinsic database (unique clade-specific marker genes were identified from ~ 13,500 bacterial and archaeal reference genomes) [51]. The generated archaeal profile was manually curated by removing those taxa with a relative abundance of < 0.01% and those without 16S rDNA recovery through metagenomic binning. We also downloaded the publicly available fecal metagenomic datasets for 6 external cohorts for comparative archaeome composition analysis, including 2 Chinese populations from Beijing (*n* = 161) and Guangzhou (*n* = 171), and 4 western populations from HMP (*n* = 239), Irish (*n* = 117), the UK (*n* = 211) and the USA (*n* = 97), under the dataset accession numbers NCBI SRA SRA045646, EBI ENA ERP023788, NIH Human Microbiome Project (https://portal.hmpdacc.org/), EBI ENA PRJEB36820, EBI ENA PRJEB39223, respectively.

### Functionality profiling of the gut archaeome

Metagenomic sequence reads were rarefied and binned to the recently established human gut archaeome catalogue (1167 genomes) [1], via *BBMap 38.22* [52]. The sequence classified as archaeal were then queried against the EGGNOG and MetaCyc databases within the HMP Unified Metabolic Analysis Network via *HUMAnN3.0* [51], for profiling the abundance of microbial metabolic pathways and other molecular functions.

### Clinical metadata and covariates of archaeome variations

We collected subject clinical metadata including ethnicity information, geography, rural versus urban residency, medication history, dietary habits, lifestyle, bowel habits, and host biological traits (Supplementary Table 2). All metadata variables were classified into the following six categories: urbanization (rural/urban residency, bath/shower frequency, duration of time residing in urban cities, education level, travel frequency, stress at work, consumption of wild foods, consumption of convenience food), ethnicity (six ethnic groups), geography (Kunming [urban] and 6 minority ethnicity residing rural regions), medication (western medicine, Chinese medicine, prebiotics/probiotics, antibiotics), dietary habit (frequency of intake of fiber-rich vegetables, meat), host biological traits (age, gender, height, BMI, bowel habit, stool consistency), and other metadata (breastfeeding, animal contact, anthropometric parameters, etc). Covariates of archaeome variation were identified by calculating the association between continuous or categorical phenotypes and species-level community ordination with *envfit* function [53] in the vegan R package (999 permutations; false discovery rate FDR<5% [54]). This function performs *manova* and linear correlations for categorical and continuous variables, respectively. Their effect sizes were pooled into the broader predefined categories, estimated with the *bioenv* function in the same package [55], which selects the combination of covariates with strongest correlation to archaeome variation (correlation between Gower distances of covariates and archaeome Bray-Curtis dissimilarity). To identify significant food item-covariate associations, pairwise chi-square test with Crammer’s V estimation and multiple-comparison adjustment (FDR) were performed. Odds ratios between food items and these metadata factors of interest (ethnicity, rural versus urban residency) were calculated by unconditional maximum likelihood estimation (Wald). *MaAsLin2* [56] R package (huttenhower.sph.harvard.edu/maaslin2) were used to identify food item-archaeal species correlations with 5% significance level (after multiple testing correction).

### Microbiome data analysis

Archaeome and bacteriome compositional data (relative abundance) were used for downstream anslyses. The abundance data was imported into *R 4.0.3*. Calculations of diversity (Shannon index), evenness (Pielou index), and richness (Chao1 index) were performed using *phyloseq* in R, based on rarefication to even sequencing depth. Centered log-ratio (CLR) transformation was applied to the relative-abundance compositional data [57, 58]. Given an observation vector of *D* taxa in a sample, **x** = [x1, x2, …x*D*], the clr transformation for the sample was obtained as follows:

$${\mathrm{X}}_{\mathrm{clr}}=\left[\log \left(\mathrm{x}1/\mathrm{G}\left(\mathbf{x}\right)\right),\log \left(\mathrm{x}2/\mathrm{G}\left(\mathbf{x}\right)\right)\dots \log \left(\mathrm{x}\mathrm{D}/\mathrm{G}\left(\mathbf{x}\right)\right)\right],$$

$$G\left(\boldsymbol{x}\right)=\sqrt[D]{x1\cdot x2\cdots xD},$$

*G*(**x**) is the geometric mean of **x**. Principal Component Analysis (PCA) were performed based on Aitchison distance of the microbiome community composition [57]. β-diversity was calculated as the Bray-Curtis dissimilarities between the archaeomes of the studied subjects. For identification of trans-domain microbial species correlations, SparCC correlation analysis was conducted in R via *SpeciEasi*. Heat maps were generated using the pheatmap R package.

### Statistical analyses

To compare differences in the configuration of the fecal archaeomes between two groups (such as rural versus urban resident), LefSE [59] analyses were performed on the Huttenhower lab Galaxy server (http://huttenhower.sph.harvard.edu/galaxy/). Between-group differences were depicted in LDA effect size and FDR adjusted *p* values. MaAsLin2 analysis was performed on the archaeome composition profile to identify ethnicity-specific and region-specific archaeal taxa (confounding factors were adjusted) where multiple comparisons were FDR adjusted. Mann-Whitney or student *t* test was performed on between-two group comparisons (on PC1, α-diversity indices, and β-diversity index, between rural versus urban; on abundance between buttered milk consumers versus non-consumers). Across-population comparisons were conducted between the base mean of each group of interest and that of all groups; statistical significance was determined by Wilcoxon rank sum test with Holm-Bonferroni adjustment of *p* values. Other statistical analyses were individually indicated at each figure where appropriate.

## Supplementary Information

**Additional file 1: Supplementary Figure 1**. Variations in the gut archaeome across healthy individuals at the species level. a, Variations in the gut archaeome at the species level across all study subjects, plotted according to the relative abundance of the fecal archaeal species. b, Species-level composition of the gut archaeome composition in the profiled Chinese population. Only the top 20 species were plotted in the pie chart. c, Prevalence of the top 3 archaeal species in human feces (*M. smithii*, *M. oralis*, and *M. stadtmanae*) among all study subjects. d, e, Relative abundance and prevalence of *Methanomassillicoccales* (an archaeal order) in the fecal archaeome of Chinese populations and Western populations. Apart from the in-house Yunnnan fecal archaeome dataset, publicly available metagenomic datasets from 2 Chinese populations from Beijing and Guangzhou, and 4 western populations from HMP, Irish, the UK and the USA were downloaded for comparative analysis. Statistical significance was determined by *one way anova* with Holm-Bonferroni adjustment of *p* values, **p<0.01.

**Additional file 2: Supplementary Figure 2**. Species association with the geographical factors, longitude (a) and altitude (b). Correlation and statistical significance were determined by *MaAsLin2* with multiple comparison adjustment by FDR. Linear regression was plotted for each correlation between archaeal species and geographical factors for easy visualization.

**Additional file 3: Supplementary Figure 3**. Geographic region-specific archaeal species. The relative abundance of *M. smithii* in the fecal archaeome of populations residing in each sampled region of Yunnan. RA, relative abundance. Statistical significance was determined by *one way anova* with Holm-Bonferroni adjustment of *p* values, *p<0.05.

**Additional file 4: Supplementary Figure 4**. Ethnicity-specific archaeal species. The relative abundance of *M. stadtmanae*, *M. oralis,* and *M. smithii* in the fecal archaeome of each sampled ethnicity in Yunnan. RA, relative abundance. Statistical significance was determined by *one way anova* with Holm-Bonferroni adjustment of *p* values, *p<0.05, **p<0.01.

**Additional file 5: Supplementary Table 1**. Demographic summary of studied healthy Chinese populations.

**Additional file 6: Supplementary Table 2**. Metadata for all study subjects.

**Additional file 7: Supplementary Table 3**. Diet record data for all study subjects.

**Additional file 8: Supplementary Table 4**. Blood biomedical measurements data for all study subjects.

**Additional file 9: Supplementary Table 5**. Significant correlations between consumption of food items and archaeal species, identified by MaAsLin2.

**Additional file 10: Supplementary Table 6**. Significant correlations between archaeal and bacterial species, identified by SparCC.

**Additional file 11: Supplementary Table 7**. Ethnicity-specific EGGNOG and MetaCyc functions in the gut archaeome.

**Additional file 12: Supplementary Table 8**. EGGNOG and MetaCyc functions enriched in the gut archaeome of rural versus urban Chinese.

## Data Availability

Sequence data have been deposited to the China National GeneBank DataBase (CNGBdb) under the accession number CNP0002547. In accordance with the China government’s policy and regulations on human genetic materials for the Chinese population, all data reported in this paper shall be shared by the lead contact upon request and joint regulatory approval from the Ministry of Science and Technology of China, Sun Yat-sen University, and Kunming Medical University.
