# Supplementary figures and images for: Landscape of the gut archaeome in association with geography, ethnicity, urbanization, and diet in the Chinese population
Source: Microbiome. 2022 Sep 13;10:147. doi: 10.1186/s40168-022-01335-7 (PMC9469561; doi:10.1186/s40168-022-01335-7)

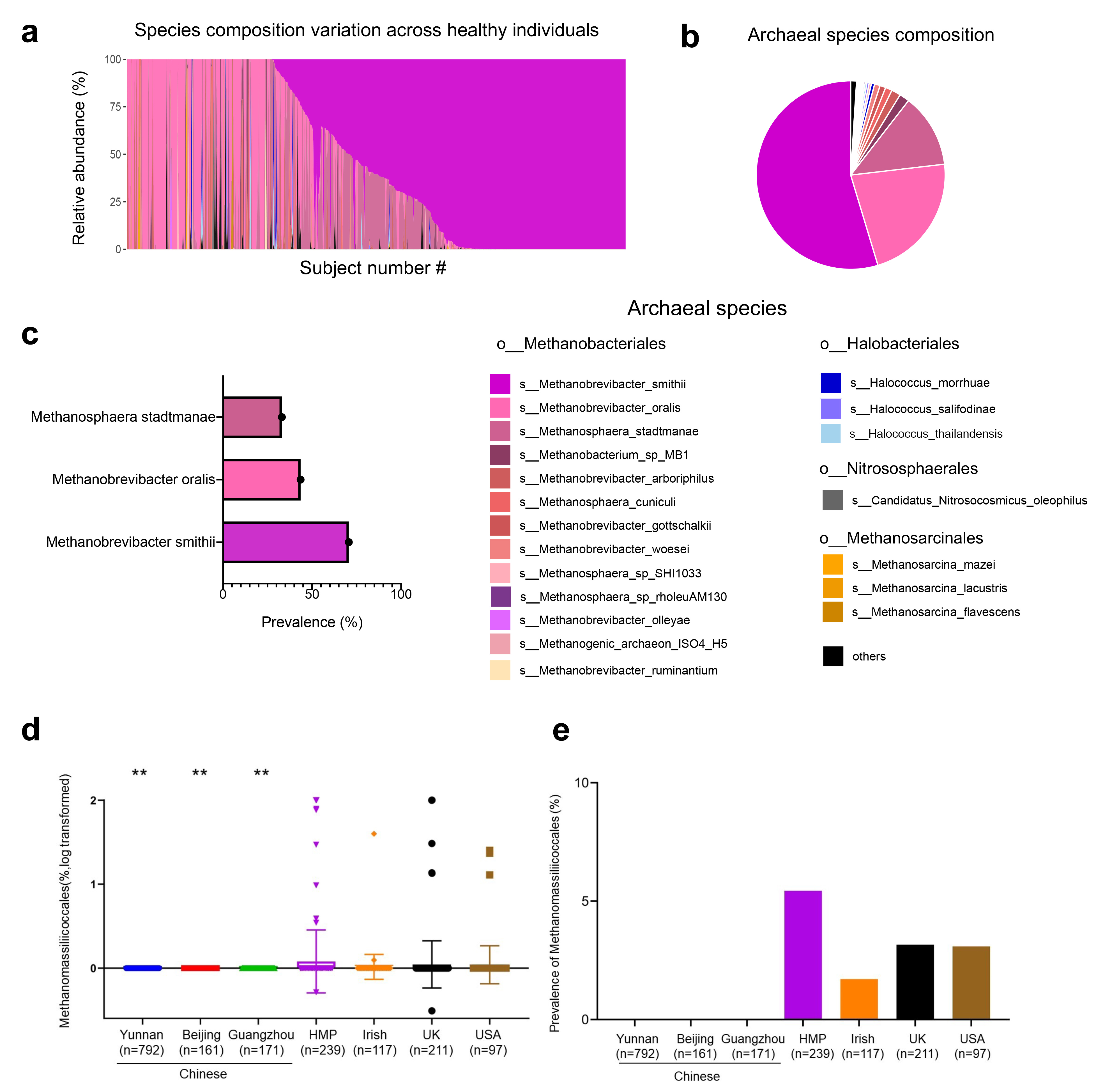

Supplement: Supplementary file 2 — Additional file 1: Supplementary Figure 1. Variations in the gut archaeome across healthy individuals at the species level. a, Variations in the gut archaeome at the species level across all study subjects, plotted according to the relative abundance of the fecal archaeal species. b, Species-level composition of the gut archaeome composition in the profiled Chinese population. Only the top 20 species were plotted in the pie chart. c, Prevalence of the top 3 archaeal species in human feces (M. smithii, M. oralis, and M. stadtmanae) among all study subjects. d, e, Relative abundance and prevalence of Methanomassillicoccales (an archaeal order) in the fecal archaeome of Chinese populations and Western populations. Apart from the in-house Yunnnan fecal archaeome dataset, publicly available metagenomic datasets from 2 Chinese populations from Beijing and Guangzhou, and 4 western populations from HMP, Irish, the UK and the USA were downloaded for comparative analysis. Statistical significance was determined by one way anova with Holm-Bonferroni adjustment of p values, **p<0.01. [file 40168_2022_1335_MOESM1_ESM.jpg]

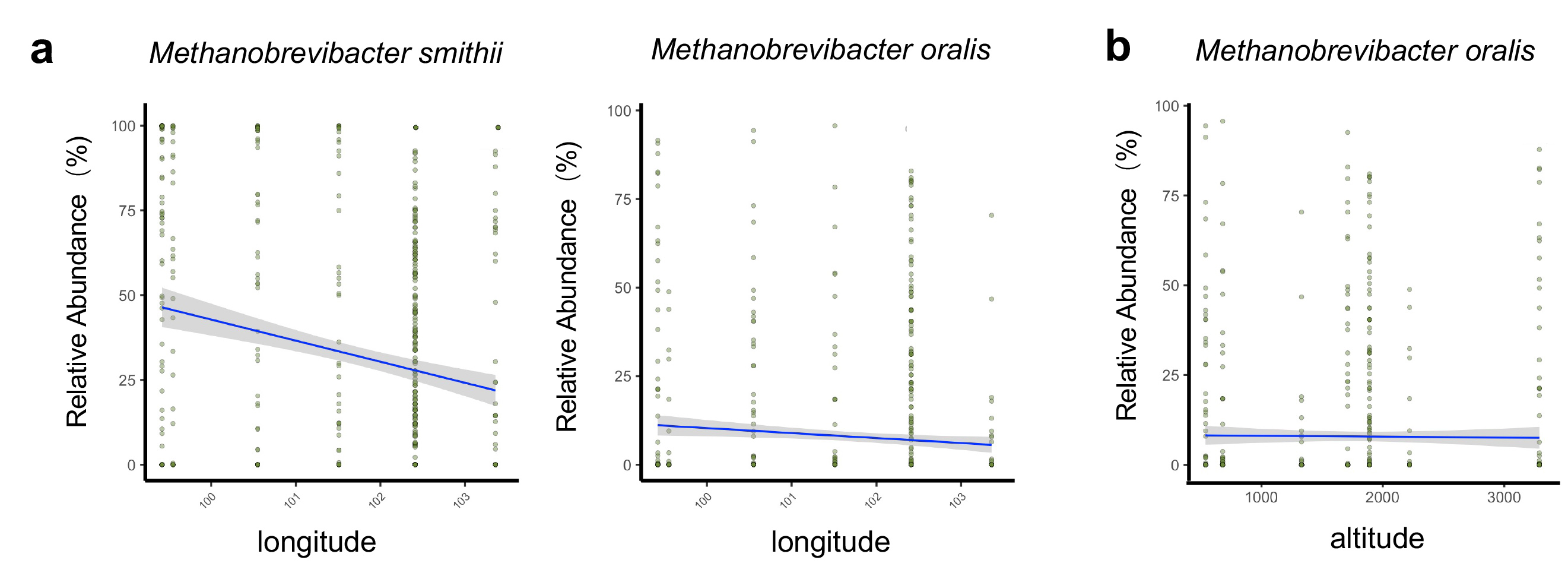

Supplement: Supplementary file 3 — Additional file 2: Supplementary Figure 2. Species association with the geographical factors, longitude (a) and altitude (b). Correlation and statistical significance were determined by MaAsLin2 with multiple comparison adjustment by FDR. Linear regression was plotted for each correlation between archaeal species and geographical factors for easy visualization. [file 40168_2022_1335_MOESM2_ESM.jpg]

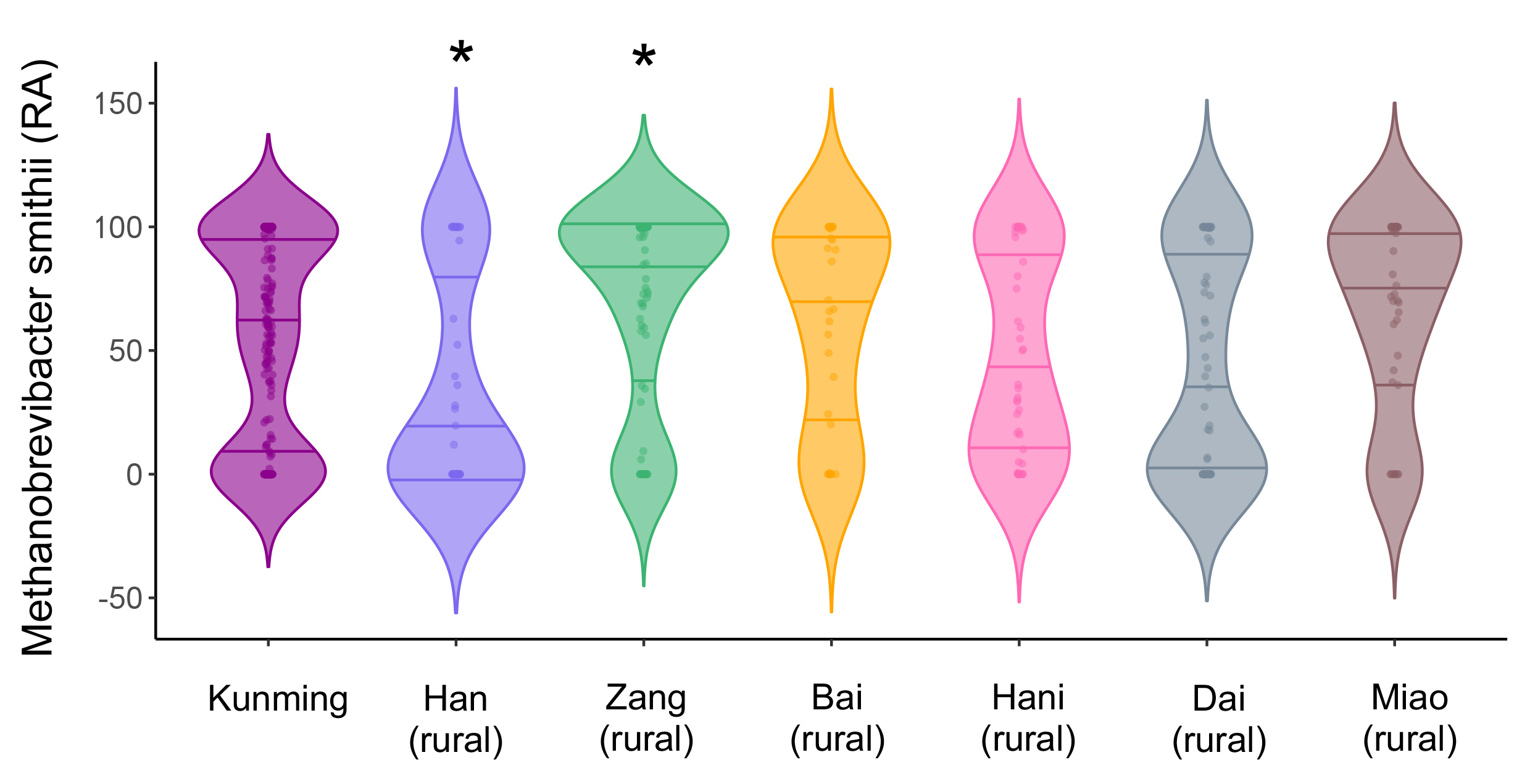

Supplement: Supplementary file 4 — Additional file 3: Supplementary Figure 3. Geographic region-specific archaeal species. The relative abundance of M. smithii in the fecal archaeome of populations residing in each sampled region of Yunnan. RA, relative abundance. Statistical significance was determined by one way anova with Holm-Bonferroni adjustment of p values, *p<0.05. [file 40168_2022_1335_MOESM3_ESM.jpg]

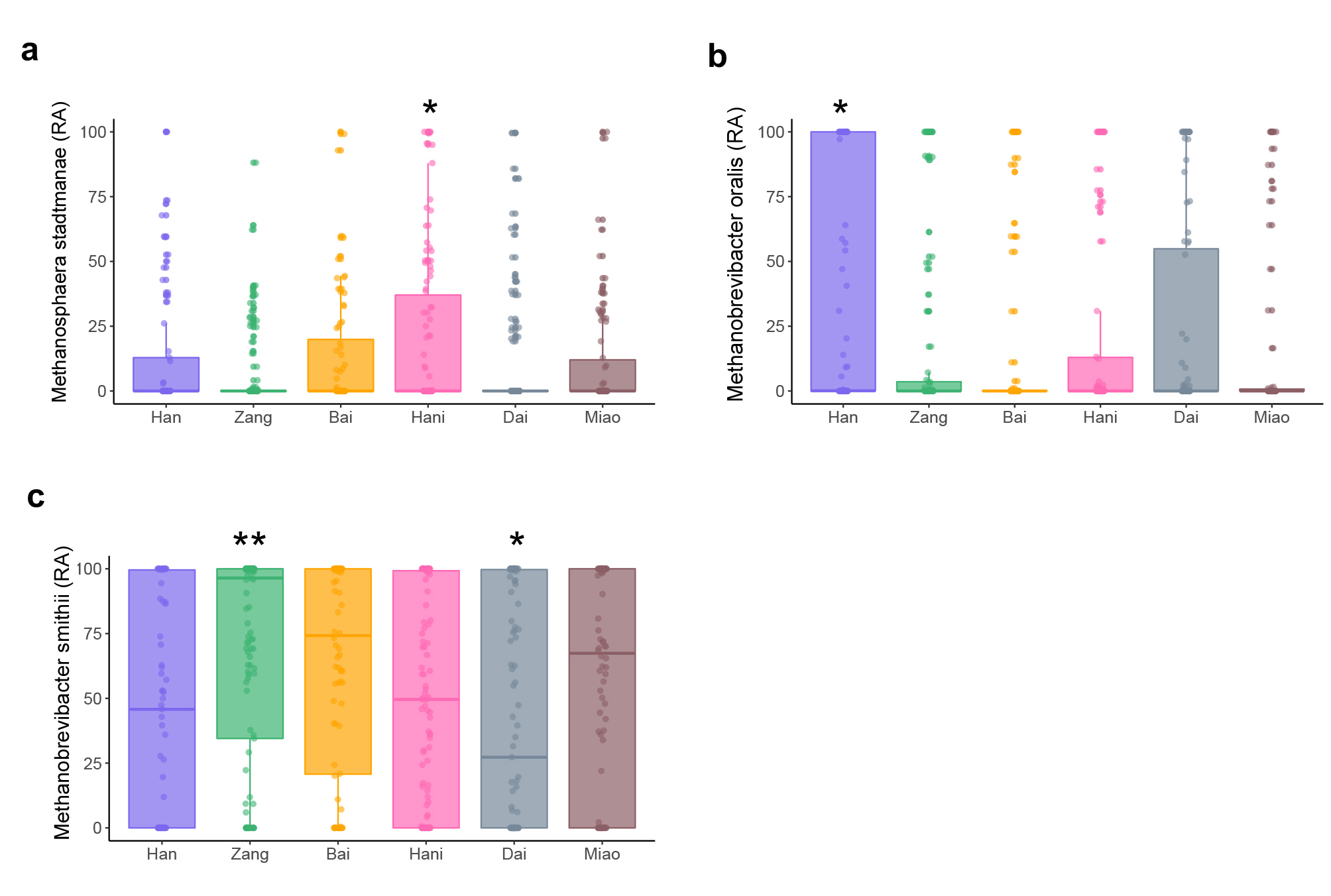

Supplement: Supplementary file 5 — Additional file 4: Supplementary Figure 4. Ethnicity-specific archaeal species. The relative abundance of M. stadtmanae, M. oralis, and M. smithii in the fecal archaeome of each sampled ethnicity in Yunnan. RA, relative abundance. Statistical significance was determined by one way anova with Holm-Bonferroni adjustment of p values, *p<0.05, **p<0.01. [file 40168_2022_1335_MOESM4_ESM.jpg]
